# Trans-segmental POEM in the Management of Esophageal Asymmetry in Achalasia: Short Outcomes from a Retrospective, Single-center Study

**DOI:** 10.1055/a-2934-6927

**Published:** 2026-10-05

**Authors:** Jintao Xiao, Xingyang Luo, Bingbing Li, Yaochong Tan, Yu Wu, Xiaowei Liu

**Affiliations:** 1 Department of Gastroenterology 159374 Xiangya Hospital Central South UniversityChangshaHunanChina; 2 Hunan International Scientific and Technological Cooperation Base of Artificial Intelligence Computer Aided Diagnosis and Treatment for Digestive DiseaseChangshaHunanChina; 3 Teaching and Research Section of Clinical Nursing 159374 Xiangya Hospital Central South UniversityChangshaHunanChina; 4 National Clinical Research Center for Geriatric Disease 159374 Xiangya Hospital Central South UniversityChangshaHunanChina

**Keywords:** endoscopy upper GI tract, motility/achalasia, POEM, quality and logistical aspects, quality management

## Abstract

**Background and Study Aims** Esophageal asymmetry has been observed in some patients with achalasia, presenting as functional or structural alterations. This study aims to report a single-center experience with the trans-segmental peroral endoscopic myotomy (T-POEM) procedure for the management of esophageal asymmetry in AC.

**Patients and Methods** Twenty-two patients with achalasia and severe dysphagia were included. Esophageal asymmetry and restrictive segments were assessed using the Eckardt score, HRM, barium esophagography, CT, EUS, and endoscopy. T-POEM was performed with targeted myotomy of tortuous segments, areas of localized muscular thickening, and localized restrictive muscular segments. This study evaluated the technical feasibility, safety, and observed short-term outcomes of T-POEM.

**Results** T-POEM was technically successful in all 22 patients (100%), with a mean procedure time of 67 min. Three patients (13.6%) experienced documented periprocedural adverse events: one developed pneumothorax with fever, and two experienced transient retrosternal pain. All three events resolved with conservative treatment, and no event required endoscopic or surgical intervention or intensive care. Clinical success, defined as an Eckardt score ≤3 at the latest available follow-up, was observed in all 22 patients. The mean Eckardt score decreased from 6.7 to 1.3 ( *P* < 0.0001) during the available short-term follow-up.

**Conclusion** In this small retrospective case series, T-POEM was technically feasible and was associated with short-term symptom improvement in selected patients with complex achalasia and marked esophageal asymmetry. These preliminary findings do not establish superiority over existing POEM techniques and require confirmation in larger prospective comparative studies.

## Introduction

Achalasia is a rare esophageal motility disorder characterized by absent esophageal peristalsis and impaired relaxation of the lower esophageal sphincter (LES). 1 In the early stages of achalasia, the esophagus generally maintains a relatively symmetric morphology before progressive dilation, tortuosity, and structural deformity occur. In such patients, peroral endoscopic myotomy (POEM) has demonstrated excellent therapeutic efficacy and technical feasibility and has been widely adopted in clinical practice. 2 3 4

Nevertheless, patients who do not receive timely intervention may progress to the advanced achalasia, while others may develop persistent or recurrent symptoms after prior treatment. These patients often exhibit marked esophageal asymmetry due to severe tortuosity, localized muscular thickening, fibrosis, or focal constrictive segments ( Fig. 1 ). Although POEM is an effective rescue treatment for advanced achalasia, creating a conventional submucosal tunnel can be technically challenging in a markedly dilated or tortuous esophagus. 5 6 7 Submucosal fibrosis may further increase procedural difficulty and the risk of mucosal injury. 8 9

**Fig. 1:**
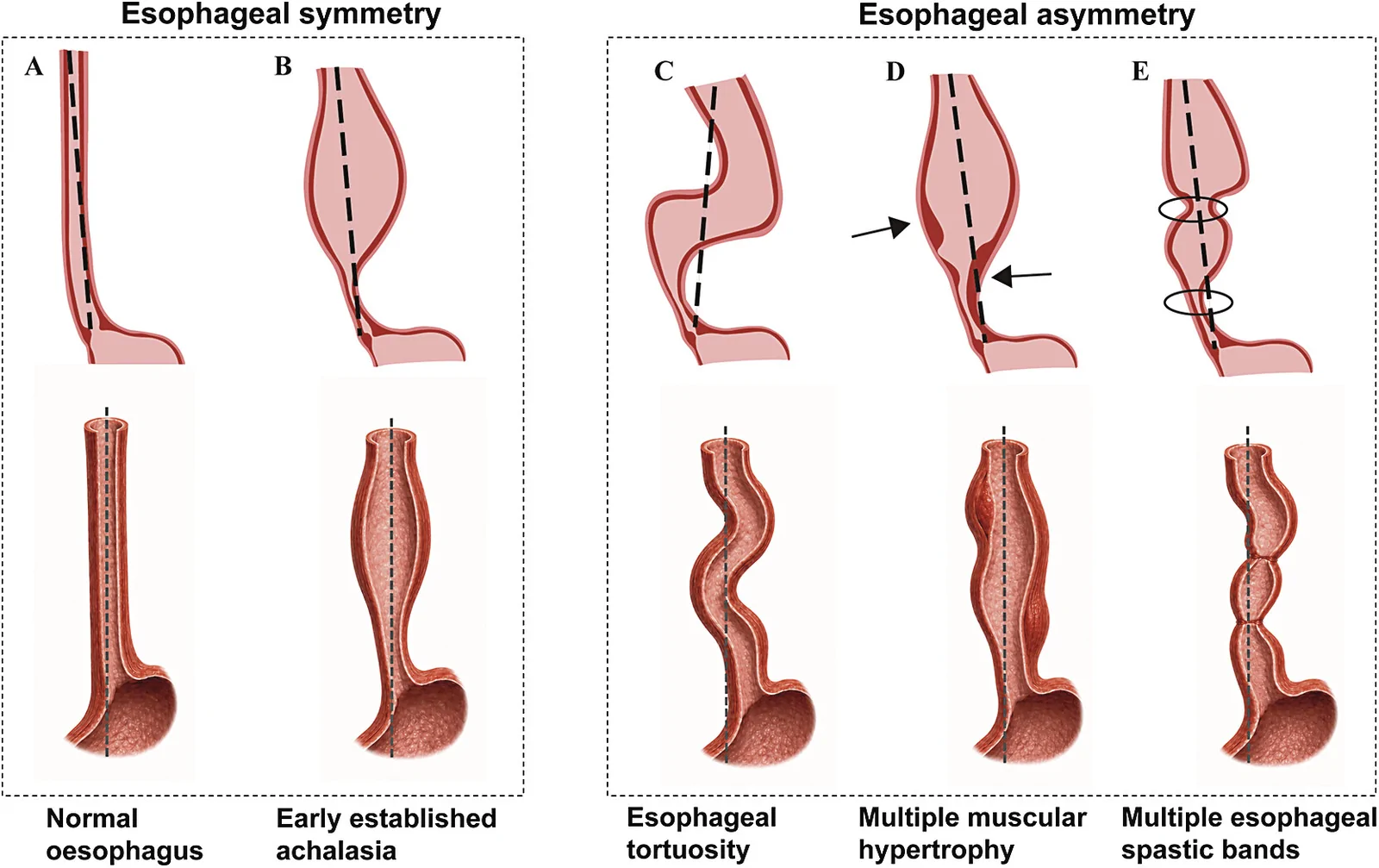
Esophageal manifestations in achalasia. In the normal esophagus ( **A** ) and in early stage achalasia ( **B** ), the lumen generally shows an approximately axisymmetric morphology; the dashed line represents the longitudinal esophageal axis. Advanced achalasia may show asymmetric morphology, including esophageal tortuosity ( **C** ), localized muscular thickening (arrow) ( **D** ), and localized restrictive muscular segments (circle) ( **E** ).

Several modified POEM techniques have been developed for patients with advanced achalasia or altered esophageal anatomy. 10 11 enables direct myotomy without creating a complete submucosal tunnel and may be particularly useful when severe submucosal fibrosis precludes conventional tunneling. 12 13 However, some patients have multiple, spatially distinct areas of tortuosity, localized muscular thickening, or focal luminal narrowing rather than obstruction limited to the LES. In these cases, a single distal myotomy may not adequately relieve all restrictive segments.

To address this complex morphology, we adopted an individualized, segment-oriented strategy termed trans-segmental POEM (T-POEM). This approach involves selective, noncontiguous myotomy targeting segments with marked tortuosity, localized muscular thickening, or focal narrowing identified by concordant preoperative and intraoperative assessment. Three procedural approaches were selected according to the distribution of target segments and the feasibility of submucosal tunneling ( Fig. 2 ). This study aimed to describe our initial experience with T-POEM in patients with technically challenging and anatomically complex achalasia.

**Fig. 2:**
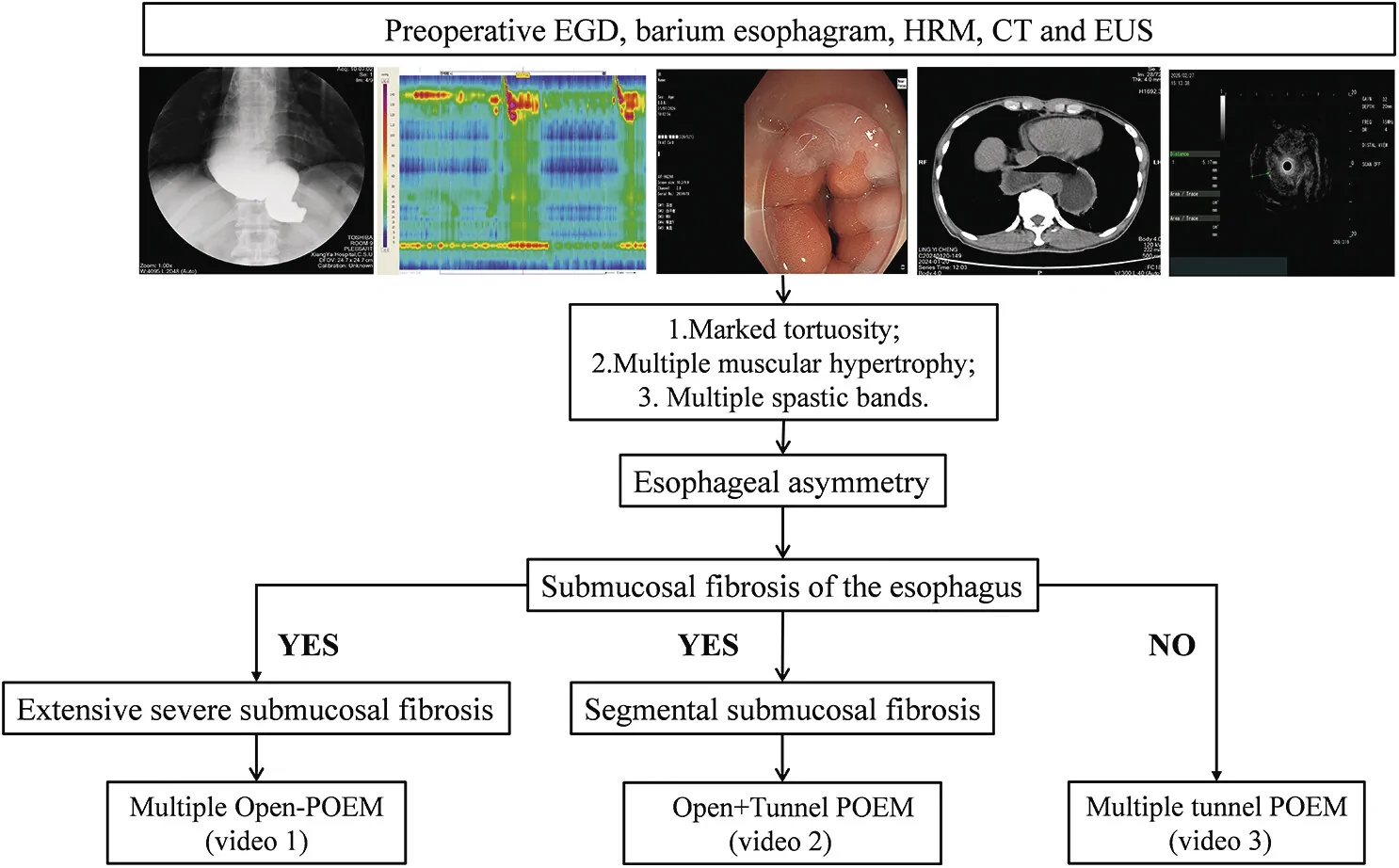
The clinical procedure of T-POEM in the management of esophageal asymmetry.

## Methods

### Study Design

This retrospective study included adults with confirmed achalasia who severe persistent or recurrent symptoms between January 2023 and March 2025. 14 Esophageal asymmetry was defined as deviation of the luminal axis accompanied by at least one of the following: (1) marked tortuosity or focal angulation; (2) asymmetric or localized muscularis propria thickening on EUS, supported by CT when available; or (3) localized restrictive muscular segments with focal narrowing or impaired opening on barium esophagography and/or endoscopy. HRM provided supportive physiological evidence when interpretable. These criteria were qualitative, with no prespecified thresholds for axis deviation, angulation, or muscle thickness. Exclusion criteria were unacceptable procedural risk due to severe comorbidities, pseudoachalasia, or severe esophagitis with submucosal inflammatory fibrosis. Follow-up included the postoperative Eckardt score, reflux-related symptoms, and available postoperative imaging. Baseline and procedural data are summarized in Tables 1 and 2 .

**Table 1:** Patient demographics and perioperative characteristics.

Demographics and perioperative characteristics	*n* (%)
Sex (female/male) ( *n* )	12/10
Age [mean range] (years)	58.2 (21–83)
Preoperative median Eckardt score	6.7
Duration of symptoms [mean range] (years)	10.2 (4–21)
Previous treatment	
Endoscopic balloon dilation	8
Heller myotomy	4
POEM	10
HRM (Chicago classification)	
I	3
II	11
III	5
HRM subtype unavailable	3
Preoperative maximum esophageal diameter (mm)	51.1 (40–68)

**Table 2:** Procedure-related parameters and clinical outcomes.

Procedure-related parameters	
Procedure time, mean (range) (min)	67 (53–82)
Incision length, mean (range) (cm)	4.9 (2–9)
Methods of T-POEM	
Multiple tunnel	6
Multiple open	10
Open-Tunnel	6
Number of incisions	
2	5
3	17
Length of postoperative hospital stay, mean (range)	6 (4–11)
Procedure-related adverse events, *n* (%)	3 (13.6)
Follow-up duration, mean (range), months	7.2 (5–9)
Postoperative median Eckardt score	1.3 (0–3)
Clinical reflux complications Symptomatic reflux only, *n* 2 Esophagitis on EGD only, *n* 1	3/22
Gastric-side myotomy length, mean (range), cm	2.7 (2–4)

Importantly, patients were informed that T-POEM was not an established standard treatment for their condition but represented one of several potential therapeutic options. All of the patients provided written informed consent after receiving a detailed explanation of the potential benefits and risks of the procedure. The study was approved by the Institutional Review Board of Xiangya Hospital.

### T-POEM Procedure



**Video 1** Multiple-open T-POEM.


**Video 2** Combined open-tunnel T-POEM.


**Video 3** Multiple-tunnel T-POEM.


Preoperative assessment included HRM, barium esophagram, CT, EUS, and EGD. Localized muscular thickening was defined as focal muscularis propria thickening on EUS relative to adjacent segments, with CT findings considered supportive. Localized restrictive muscular segments were identified by concordant evidence of focal narrowing, angulation, or impaired opening on barium esophagography and/or endoscopy, supported by HRM when interpretable. Submucosal fibrosis was suspected from prior intervention or endoscopic scarring but confirmed intraoperatively. Extensive fibrosis was defined as dense adhesions, loss of the normal submucosal plane after injection and dissection, and inability to create a conventional tunnel without an unacceptable risk of mucosal injury.

Three procedural strategies were used: (1) multiple-tunnel T-POEM, involving separate submucosal tunnels and targeted myotomies at two or more noncontiguous restrictive segments when a safe submucosal plane could be established at each site; (2) multiple-open T-POEM, involving direct myotomy through separate mucosal openings at multiple target segments when extensive fibrosis or distorted anatomy made conventional tunneling unsafe or impractical; and (3) combined open-tunnel T-POEM, using tunneling at segments with a preserved submucosal plane and an open approach at separate segments with extensive fibrosis or unfavorable anatomy. The number and extent of myotomies were individualized according to the number and distribution of discrete targets identified by multimodal assessment, with the fewest segments necessary to relieve the identified restrictions treated. This approach represented an institutional operational framework rather than a validated selection algorithm ( Fig. 2 ).

All procedures were performed in the endoscopy unit with patients under general anesthesia with endotracheal intubation and in the left lateral position. A high-definition gastroscope (Olympus-HQ290, Tokyo, Japan) fitted with a transparent cap was used, with carbon dioxide insufflation throughout. A electrosurgical generator and a DualKnife (Olympus-KD650L, Tokyo, Japan) were used for mucosal incision, submucosal dissection, and myotomy. After submucosal injection and mucosal incision, submucosal dissection was performed, followed by selective full-thickness myotomy extending across the gastroesophageal junction. The location of each mucosal incision was individualized according to the orientation of any previous myotomy, the distribution of submucosal fibrosis, the direction and severity of esophageal tortuosity, and the location of the target restrictive segment. In patients with prior Heller myotomy or POEM, an alternative orientation was preferentially selected to avoid the previous myotomy tract and areas of dense fibrosis. After prior pneumatic dilation, because no myotomy plane required avoidance, the incision site was determined primarily by esophageal anatomy and the anticipated route to the target segment. In the tunneling approach, the mucosal entry was closed with metal clips. For procedures incorporating an open approach, the exposed mucosal defect was intentionally left unclosed to avoid closure under tension and to allow drainage. Gastric decompression using a nasogastric tube was routinely performed. All procedures were performed by experienced POEM operators using a tunneling approach, an open method, or a combined open-tunnel approach ( **Videos 1** **–** **3** ).

### Follow-up

Patients were routinely hospitalized after T-POEM. Broad-spectrum antibiotics were administered for 3–5 days. A soft diet was recommended for 1–2 weeks, after which patients gradually resumed a normal diet. Follow-up was scheduled at 1, 3, 6, and 12 months by outpatient visit or telephone interview. The Eckardt score was assessed at each follow-up, with its four components scored from 0 to 3. The latest available postoperative score was used for the primary analysis. In addition, barium esophagram, EUS and EGD were planned at 6 months and annually thereafter to assess esophageal morphology and reflux esophagitis.

### Outcomes

The primary outcome was clinical success, defined as an Eckardt score ≤3 at the latest postoperative follow-up. Secondary objectives were to document the procedure-related parameters such as operation time, incision and myotomy lengths, hospital stay, adverse events, changes in esophageal morphology, and postoperative reflux-related outcomes. All patients with an available postoperative Eckardt score were included in the primary analysis.

### Statistical Analysis

Continuous variables were assessed for normality and are presented as mean ± SD or median (interquartile range), as appropriate. Paired continuous variables were compared using the paired Student’s *t* test or Wilcoxon signed-rank test, depending on data distribution. All tests were two-sided, with *P* < 0.05 considered statistically significant. Analyses were performed using SPSS version 22.0 (IBM Corp., Armonk, NY, USA).

## Results

### Patient Characteristics at Baseline

As shown in Table 1 , 22 patients were included, comprising 12 men and 10 women, with a mean age of 58.2 years (SD, 14.1). The mean preoperative Eckardt score was 6.7 (SD, 1.8), and the mean disease duration was 10.2 years (SD, 5.4). All patients had undergone prior achalasia treatment: pneumatic dilation in 8, Heller myotomy in 4, and conventional POEM in 10. All had persistent or recurrent symptoms after these treatments. Preoperative HRM enabled Chicago Classification subtyping in 19 patients: 3 had type I, 11 had type II, and 5 had type III achalasia. In the remaining three patients, severe esophageal dilation and tortuosity prevented passage of the manometry catheter across the gastroesophageal junction, precluding complete HRM and formal subtype assignment. Their HRM subtype was therefore recorded as unavailable, although the diagnosis of achalasia had been established. The mean maximum esophageal diameter was 51.1 mm (SD, 7.6).

### Procedure

As shown in Table 2 , technical success was achieved in all 22 patients (100%).The mean procedure time was 67 min (SD, 11.3). Five patients undergone two incisions and 17 patients had three incisions. The mean myotomy length was 4.9 cm (SD, 1.9), including a mean gastric extension of 2.7 cm (range, 2–4 cm). Periprocedural adverse events occurred in three patients (13.6%). One developed pneumothorax and fever, which resolved with conservative treatment, and two experienced transient retrosternal pain that resolved with analgesics. No patient required endoscopic or surgical intervention or intensive care. The mean postoperative hospital stay was 6.4 days (SD, 1.7).

### Clinical Outcomes

Clinical success was achieved in all patients (100%) at the latest follow-up. The mean follow-up duration was 7.2 months (range, 4–12 months). The mean Eckard score decreased from 6.7 (SD, 1.8) before T-POEM to 1.3 (SD, 0.9) after T-POEM ( *P* < 0.0001) ( Fig. 3 ). Among patients with available postoperative imaging, barium esophagography and endoscopy showed reduced esophageal tortuosity and focal luminal narrowing compared with baseline ( Table 3 ). Representative paired images are shown in Fig. 4 . During periodic follow-up, two patients developed reflux symptom at 3 and 6 months, respectively, both of which resolved after 2 months of proton pump inhibitor therapy.

**Fig. 3:**
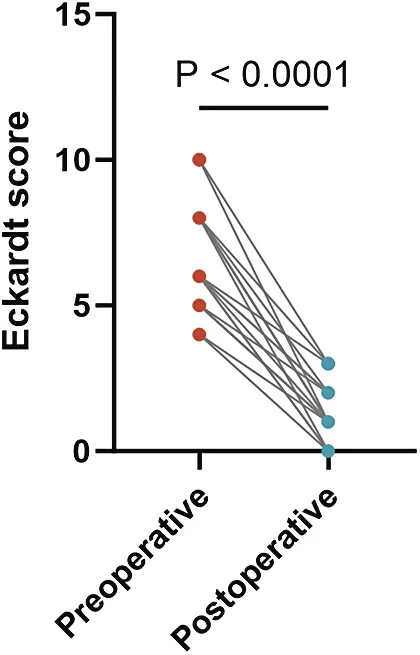
Individual paired Eckardt scores before and after T-POEM in all 22 patients.

**Table 3:** Specific changes in esophageal symmetry before and after T-POEM.

Variable	Before T-POEM *n* (%)	After T-POEM *n* (%)
Esophageal tortuosity	13 (59.1%)	2 (9.1%)
Multiple sites of localized muscular thickening	3 (13.6%)	0 (0%)
Multiple localized restrictive muscular segments	2 (9.1%)	0 (0%)
Esophageal tortuosity with localized muscular thickening	3 (13.6%)	1 (4.6%)
Esophageal tortuosity with localized restrictive muscular segments	1 (4.6%)	0 (0%)

**Fig. 4:**
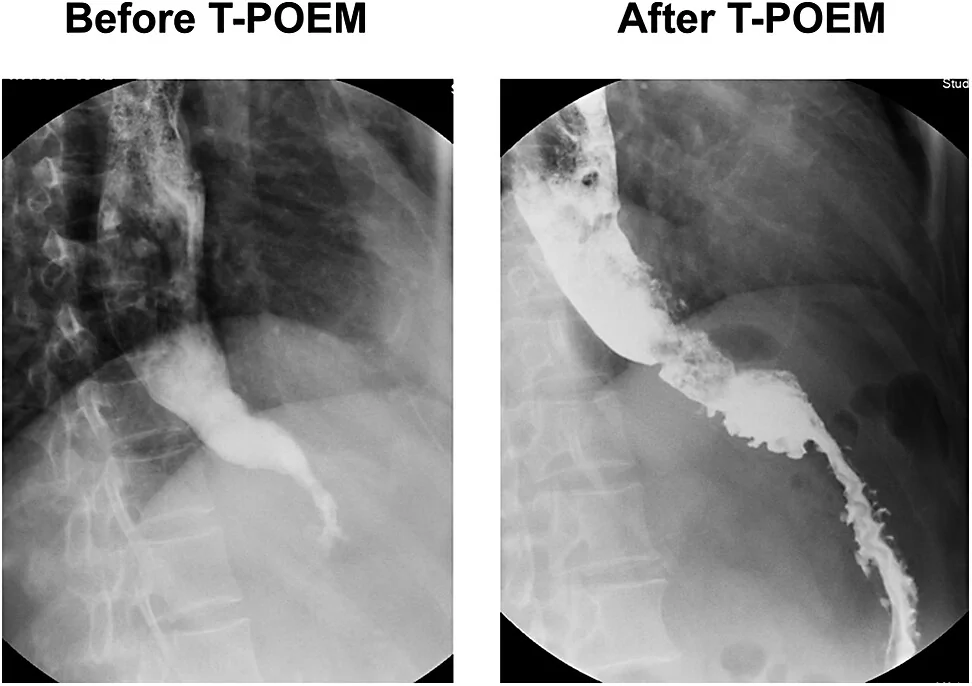
Representative preoperative and postoperative objective imaging findings after T-POEM.

## Discussion

Over years of technical development, several modified POEM approaches have been introduced for patients with advanced achalasia or difficult esophageal anatomy. Open POEM (O-POEM), which permits direct myotomy without creation of a complete submucosal tunnel, may be useful when severe submucosal fibrosis precludes conventional tunneling. However, its use in advanced achalasia has been described mainly in case reports. 15 Moreover, a single distal myotomy may not adequately relieve symptoms in patients with multiple spatially separate areas of anatomical distortion or functional restriction. In this study, T-POEM was defined as an individualized strategy involving selective myotomy of multiple noncontiguous esophageal segments rather than a single continuous myotomy.

Esophageal asymmetry has been recognized in previous physiological and imaging studies. The LES exhibits both axial and circumferential pressure asymmetry, which may be related to its muscular morphology and anatomical configuration. 16 Advanced achalasia may also present with nonuniform dilation, tortuosity, and contour abnormalities on barium esophagography, consistent with morphological asymmetry. 17 However, strategies for systematically identifying and treating these spatially distinct abnormalities in patients with achalasia remain poorly defined. Importantly, T-POEM is primarily a treatment-planning strategy. Multiple noncontiguous target segments are identified according to concordant evidence of anatomical distortion, localized muscular thickening, focal restriction, and local tunneling feasibility. A tunnel, open, or combined approach is then selected for each segment. Thus, the distinguishing feature of T-POEM is not a novel dissection or myotomy technique but the individualized mapping and treatment of spatially separate restrictive segments using established POEM approaches. We do not suggest that T-POEM represents the first use of multiple or open myotomies or that it is superior to previously described modified POEM techniques.

The extent of gastric myotomy requires a balance between adequate relief of gastroesophageal junction obstruction and the risk of postoperative reflux. Current recommendations generally support a gastric extension of 2–3 cm beyond the gastroesophageal junction. 18 Randomized evidence also suggests that gastric myotomy length may affect reflux severity, although not necessarily its overall incidence. 19 In our cohort, the mean gastric extension was 2.7 cm (range, 2–4 cm). Extension beyond 3 cm was reserved for selected patients with substantial residual obstruction after prior treatment and was not routinely performed. A 4-cm extension should therefore be considered an individualized technical decision for selected complex cases rather than a generally recommended approach.

Nonetheless, the study has several limitations. First, its retrospective, single-center design and highly selected population introduce selection bias and limit generalizability. Second, the small sample size and absence of a control group preclude definitive conclusions regarding efficacy; therefore, the observed clinical success in all 22 patients should be interpreted cautiously. Third, follow-up was short and variable, and not all patients had completed every scheduled assessment. In addition, the operational definitions and procedural selection framework were not externally validated. No prespecified quantitative thresholds were used for esophageal axis deviation, angulation, or muscularis propria thickness, and some assessments remained operator dependent. Larger prospective controlled studies incorporating quantitative definitions, standardized follow-up, and objective outcome measures are needed to validate patient selection and determine the efficacy and durability of T-POEM. In conclusion, T-POEM may provide a feasible treatment-planning strategy for selected patients with refractory achalasia and complex esophageal asymmetry. It is unlikely to be necessary for most treatment-naïve patients with early stage achalasia but may be considered in patients with persistent or recurrent symptoms accompanied by marked tortuosity, localized muscular thickening, fibrosis, or multiple restrictive segments. Further studies are required to define appropriate indications, segmentation criteria, and the optimal number and extent of treated segments. Comparative studies should also evaluate the safety and effectiveness of conventional POEM, O-POEM, and T-POEM across different operators and centers before broader adoption.
